# Misinformation can prevent the suppression of epidemics

**DOI:** 10.1098/rsif.2021.0668

**Published:** 2022-03-30

**Authors:** Andrei Sontag, Tim Rogers, Christian A. Yates

**Affiliations:** Department of Mathematical Sciences, University of Bath, Bath BA2 7AY, UK

**Keywords:** epidemic modelling, awareness spread, heterogeneous population

## Abstract

The effectiveness of non-pharmaceutical interventions, such as mask-wearing and social distancing, as control measures for pandemic disease relies upon a conscientious and well-informed public who are aware of and prepared to follow advice. Unfortunately, public health messages can be undermined by competing misinformation and conspiracy theories, spread virally through communities that are already distrustful of expert opinion. In this article, we propose and analyse a simple model of the interaction between disease spread and awareness dynamics in a heterogeneous population composed of both trusting individuals who seek better quality information and will take precautionary measures, and distrusting individuals who reject better quality information and have overall riskier behaviour. We show that, as the density of the distrusting population increases, the model passes through a phase transition to a state in which major outbreaks cannot be suppressed. Our work highlights the urgent need for effective interventions to increase trust and inform the public.

## 1. Introduction

Non-pharmaceutical interventions, such as mask-wearing and social distancing, are important tools to tackle the spread of emergent pandemic diseases [1]. They can be used to slow the progress of an outbreak or suppress it completely if carefully implemented and strictly adhered to [2,3]. There are, however, substantial drawbacks, including immediate economic damage to certain industries and longer-term negative effects of social isolation on the wellbeing of individuals. Beyond any rational cost–benefit analysis, these measures also evoke a strong emotional response. The concept of a sudden government demand for people to change their behaviour and limit social interaction in the face of an unseen enemy is at odds with prevailing western liberal ideologies of the importance of individual freedoms. Unsurprisingly, these policies do not enjoy universal support and public perceptions are a key consideration in deciding implementation details—it has been widely reported that the UK government delayed action in spring 2020 for fear that ‘behavioural fatigue’ would limit public adherence to social distancing rules [4].

Much of the effectiveness of social distancing strategies relies upon the behaviours of individuals; how conscientiously they follow advice and how successfully they encourage others to do the same. Many factors affect the degree to which individuals will change their behaviours: perception of the risk to themselves and to others; quality and availability of information; trust in the government and/or scientific establishment; the attitudes of their social contacts to these issues. Importantly, there is considerable feedback between these factors and the progression of the epidemic itself [5]. Previous modelling studies have examined the interplay between risk perception (modelled as an information spreading process), behaviour and epidemic dynamics. Methodologies applied include: game theory models for social distancing [6], opinion dynamics for behavioural change [7], models of fear of infection and fear of the control [8], SIS-like models with aware categories on networks where behaviour changes according to health status and risk perception [9], SIR models with aware and unaware states [10] and awareness-driven reduction in contacts [11].

Of particular interest is the work by Funk *et al.* [12] investigating the interplay between behavioural change and disease spread through a stochastic SIR-like model [13] coupled with an awareness model in which individuals transmit information (awareness) through a contact-based network. The authors characterize the dynamics of information spread by the following two event types: (1) *transmission of awareness*, occurring when an individual encounters someone with more up-to-date information, and (2) *fading of awareness*, which describes gradual relaxation back to a low-awareness state. The feedback between disease and information dynamics occurs through the generation of new information by infected individuals when they realize their condition, after which they and their contacts may adopt safer behaviours to limit further spread of the infection. In [14] (and similarly in independent work of [15]) a simplified version of this model was explored with only two states of aware–unaware individuals, in which it was shown that awareness-driven behaviour change can alter the outbreak conditions and can make it impossible for a disease to establish itself in the population.

The works discussed above share common (often unstated) assumptions about the populations they describe. They model homogeneous populations of individuals that believe in the existence of the disease, are prepared to alter their behaviour to reduce transmission, and have been correctly informed how to do so. Recent evidence sadly shows the extent to which these assumptions fail [16–20].

The current COVID-19 pandemic represents a major case study for the impact of mis/disinformation on the epidemic trajectory itself [21–23]. During an epidemic, the fast dissemination and adoption of public health practices plays a fundamental role in curbing disease spread. The advent of social media and its increased adoption in recent years has facilitated peer-to-peer dissemination of news and information. Despite its potential for benefit, the misuse of social media and the uncontrolled dissemination of unverified material has led to a so-called infodemic, jeopardizing the adoption of public health policies and practices [24]. The World Health Organization (WHO) defines an infodemic as ‘an overabundance of available information—accurate or not—that hampers people’s search for trustworthy and reliable sources’ [25].

Infodemics pose a serious challenge for public health [26,27], potentially causing panic, fear and distrust [28]. The spread of disinformation regarding the COVID-19 pandemic has had devastating consequences. For instance, it may have led to the adoption of unscientific treatments that caused more harm than good [29,30]. Furthermore, belief in conspiracy theories has been linked to a decreased in compliance with precautionary measures and distrust in government officials, suggesting that misinformation may contribute to increasing the severity of the pandemic [31–33]. Past research has pointed to ‘trust in the government’ as closely related to vaccine acceptance and compliance with health recommendations [34–36]. Additionally, it has been observed that people exposed to misinformation have reduced intention to be vaccinated [37–39].

In this study, we explore the effects of awareness spread on epidemics in a heterogeneous population. Awareness is to be understood as the possession of information about the outbreak that spurs one to use non-pharmaceutical interventions (NPIs) to reduce disease transmission. We extend the established model of [12] to include a subset of individuals who are distrustful of official advice, susceptible to misinformation, and more likely to engage in risky behaviours. We show that the presence of such dynamics can substantially reduce the effectiveness of behaviour-change-based epidemic intervention strategies. We find a new dynamical threshold that corresponds to a transition between regimes of successful disease suppression and large outbreaks. In the limit of fast information spread, a theoretical estimate of the critical parameters for this transition is obtained. This non-trivial benefit gained from increasing the trust of the population and the adoption of better NPIs highlights the importance of developing more efficient policies to reduce the spread of misinformation.

The article is organized as follows. In §2, we propose a model of epidemics with behaviour change feedback in a heterogeneous population composed of trusting and distrusting individuals. Results from the modelling approach are presented in §3; we discuss the observed phase transition in §3.1 and derive a theoretical estimate for the fast spread of information limit in §3.2. We conclude with a brief discussion of our results in §4.

## 2. The model

The model merges information and disease dynamics to investigate the effects of awareness-driven behavioural changes on epidemics. As in [12], the transmission of information (epidemic awareness) occurs as a contact-based process rather than through media outlets. These interactions include day-to-day conversations and social media contact, but exclude radio and television coverage. We extend the model by Funk *et al*. [12] with the introduction of a heterogeneous population composed of (i) *trusting* individuals who seek information of higher quality and take self-initiated precautionary measures, and (ii) *distrusting* individuals, who tend to disbelieve new data in favour of older or distorted information. The terms *trust* and *distrust* are used here and throughout to characterize the individuals’ belief or disbelief of data and guidelines provided by reliable sources, such as the WHO, UK’s National Health Service (NHS) and medical experts (virologists, epidemiologists and public health scholars).

Similarly to [12], we consider a population of fixed size *N* and SIR dynamics for disease spread [40]. In the SIR model, the population is compartmentalized into three disease states: susceptible *S*, infected *I* and removed *R*. We distinguish subpopulations in our model by two indexes giving the behavioural group to which they belong, i.e. trusting (*T*) or distrusting (*D*), and the quality, $i\in N_{0}$, of the information they possess, indicating lower or higher levels of awareness of the disease and correspondingly a greater propensity to act to prevent its spread. Awareness level decreases with increasing index *i* so that, for example, an individual in the group *S*_*T*,0_ is a susceptible member of the trusting population possessing the best awareness. Once an infected individual is diagnosed, their level of awareness is refreshed back to zero.

The two groups of trusting and distrusting individuals are distinguished by the way they obtain information. Trusting individuals of any disease state *X* ∈ {*S*, *I*, *R*} with awareness level *j*, *X*_*T*,*j*_, will accept new information when they encounter individuals of any group with information of better quality *i*, i.e. *X*_*T*,*j*_ + *X*_*Y*,*i*_ → *X*_*T*,*i*+1_ + *X*_*Y*,*i*_, where *i* < *j* and *Y* can be either trusting (*T*) or distrusting (*D*). The ‘+1’ in the awareness subscript represents a loss in quality when the information is passed from one individual to another. Hence, trusting individuals possess the same information dynamics as assigned to all individuals in [12]. By contrast, distrusting individuals *X*_*D*,*j*_ get information from worse-informed individuals, i.e. *X*_*D*,*j*_ + *X*_*Y*,*i*_ → *X*_*D*,*i*+1_ + *X*_*Y*,*i*_, where *i* > *j*. Parameters *α*_*T*_ and *α*_*D*_ control the encounter rates for each subpopulation.

Besides information transmission, we also assume a *fading* effect. Due to the fading effect, awareness fades if not refreshed, increasing the individual’s index by 1, i.e. *X*_*i*_ → *X*_*i*+1_. This occurs at a rate *λ*. This dynamic accounts for reluctance of the population to continue with inconvenient measures when they do not perceive immediate risk. The level of awareness of an individual, *i*, indicates how many times the acquired information has faded or been transmitted since it has been generated by an infection.

Despite the probabilistic nature of the interactions just described, the stochastic model does not offer significant improvement over a simpler ODE model in the large population size limit. Hence, ignoring any disease dynamics for the moment, the described information dynamics lead to the following equations representing the changes of population size at each information level *k* for each group of trusting and distrusting individuals and any disease state *X* ∈ {*S*, *I*, *R*},

2.1 $$\begin{array}{rl} \frac{dX_{T,k}}{dt} & =-\frac{\alpha_{T}}{N}X_{T,k}\sum_{i=0}^{k-2}(X_{T,i}+X_{D,i})\\ & \;+\frac{\alpha_{T}}{N}(X_{T,k-1}+X_{D,k-1})\sum_{i=k+1}^{\infty }X_{T,i}-\lambda X_{T,k}+\lambda X_{T,k-1} \end{array}$$

and

2.2 $$\begin{array}{rl} \frac{dX_{D,k}}{dt}= & -\frac{\alpha_{D}}{N}X_{D,k}\sum_{i=k+1}^{\infty }(X_{T,i}+X_{D,i})\\ \; & +\frac{\alpha_{D}}{N}(X_{T,k-1}+X_{D,k-1})\sum_{i=0}^{k-2}X_{D,i}-\lambda X_{D,k}+\lambda X_{D,k-1}, \end{array}$$

which are valid for *k* ∈ {0, 1, …} if we take *X*_*Y*,*j*_ = 0 for *j* < 0. The first term in equation (2.1) corresponds to individuals at the information level *k* moving to a better quality of information after interacting with either trusting or distrusting better-informed individuals. The second term corresponds to trusting individuals with worse information that interact with individuals at the level *k* − 1, acquiring information of quality *k*. The last two terms describe the changes due to the fading effect. Similarly, the first term in equation (2.2) gives the changes due to distrusting individuals with information quality *k* obtaining worse quality of information. The second term is the gain due to individuals with better information quality receiving information from level *k* − 1. The final terms again describe the changes due to information fading.

The above awareness model, given by equations (2.1) and (2.2), describes changes in distribution of the population in the awareness levels due to transmission and fading once some level of disease awareness is present in the population. Fresh information is generated by disease interactions, whilst different levels of awareness lead to lower or higher adoption of non-pharmaceutical interventions. These two dynamics create mutual feedback between information dynamics, behavioural changes and disease spread which we describe next.

Denoting *I*_*i*_ = *I*_*T*,*i*_ + *I*_*D*,*i*_, *t*_*i*_ = *S*_*T*,*i*_ + *I*_*T*,*i*_ + *R*_*T*,*i*_, and *d*_*i*_ = *S*_*D*,*i*_ + *I*_*D*,*i*_ + *R*_*D*,*i*_, we obtain the following set of ordinary differential equations to describe the changes in the subpopulations due to disease and behaviour dynamics,

2.3 $$\begin{array}{rll} \frac{dS_{T,k}}{dt} & =-\frac{S_{T,k}}{N}\sum_{i=0}^{\infty }I_{i}\cdot (1-\rho^{i})(1-\rho^{k})\beta -\frac{\alpha_{T}}{N}S_{T,k}\sum_{i=0}^{k-2}(t_{i}+d_{i}) & \left(2.3\right)\\ & \;-\lambda S_{T,k}+\lambda S_{T,k-1}+\frac{\alpha_{T}}{N}(t_{k-1}+d_{k-1})\sum_{i=k+1}^{\infty }S_{T,i}, \end{array}$$

2.4 $$\begin{array}{rll} \frac{dS_{D,k}}{dt} & =-\frac{S_{D,k}}{N}\sum_{i=0}^{\infty }I_{i}\cdot (1-\rho^{i})(1-\rho^{k})\beta -\frac{\alpha_{D}}{N}S_{D,k}\sum_{i=k+1}^{\infty }(t_{i}+d_{i}) & eq_{s}d\\ & \;-\lambda S_{D,k}+\lambda S_{D,k-1}+\frac{\alpha_{D}}{N}(t_{k-1}+d_{k-1})\sum_{i=0}^{k-2}S_{D,i}, \end{array}$$

2.5 $$\frac{dI_{T,0}}{dt}=-\sigma I_{T,0}+\omega \sum_{i=1}^{\infty }I_{T,i}-\lambda I_{T,0},$$

2.6 $$\begin{array}{rll} \frac{dI_{T,k}}{dt} & =\frac{S_{T,k}}{N}\sum_{i=0}^{\infty }I_{i}\cdot (1-\rho^{i})(1-\rho^{k})\beta -\sigma I_{T,k}-\frac{\alpha_{T}}{N}I_{T,k}\sum_{i=0}^{k-2}(t_{i}+d_{i}) & eq_{i}t\\ & \;-\lambda I_{T,k}+\lambda I_{T,k-1}+\frac{\alpha_{T}}{N}(t_{k-1}+d_{k-1})\sum_{i=k+1}^{\infty }I_{T,i}-\omega I_{T,k}, \end{array}$$

2.7 $$\frac{dI_{D,0}}{dt}=-\sigma I_{D,0}+\omega \sum_{i=1}^{\infty }I_{D,i}-\frac{\alpha_{D}}{N}I_{D,0}\sum_{i=1}^{\infty }(t_{i}+d_{i})-\lambda I_{D,0},$$

2.8 $$\begin{array}{rll} \frac{dI_{D,k}}{dt} & =\frac{S_{D,k}}{N}\sum_{i=0}^{\infty }I_{i}\cdot (1-\rho^{i})(1-\rho^{k})\beta -\sigma I_{D,k}-\frac{\alpha_{D}}{N}I_{D,k}\sum_{i=k+1}^{\infty }(t_{i}+d_{i}) & \\ & \;-\lambda I_{D,k}+\lambda I_{D,k-1}+\frac{\alpha_{D}}{N}(t_{k-1}+d_{k-1})\sum_{i=0}^{k-2}I_{D,i}-\omega I_{D,k}, \end{array}$$

2.9 $$\begin{array}{rll} \frac{dR_{T,k}}{dt} & =\sigma I_{T,k}-\frac{\alpha_{T}}{N}R_{T,k}\sum_{i=0}^{k-2}(t_{i}+d_{i})-\lambda R_{T,k}+\lambda R_{T,k-1} & eq_{r}t\\ & \;+\frac{\alpha_{T}}{N}(t_{k-1}+d_{k-1})\sum_{i=k+1}^{\infty }R_{T,i}, \end{array}$$

2.10 $$\begin{array}{rl} \mathrm{and}\;\;\frac{dR_{D,k}}{dt} & =\sigma I_{D,k}-\frac{\alpha_{D}}{N}R_{D,k}\sum_{i=k+1}^{\infty }(t_{i}+d_{i})-\lambda R_{D,k} \end{array}$$

where *β* is the transmission rate in the absence of any protective measures, *σ* is the recovery rate, and *ρ* is a fixed parameter which measures the effectiveness of non-pharmaceutical interventions, 0 < *ρ* < 1. The first terms in equations (2.3), (2.4), (2.6) and (2.8) correspond to new infections of susceptible individuals. In contrast with the model [12], we assume that disease transmission is reduced by both infected and susceptible individuals reducing their contact rates, hence the factor (1 − *ρ*^*i*^)(1 − *ρ*^*k*^) in the transmission rates. In this case, infectious individuals at information level 0 do not infect any susceptibles during the period they are fully aware of their condition. This is the case for diseases for which self-isolation is imposed after the detection of the disease—assuming this occurs before the individual recovers.

The second link between disease and awareness dynamics is obtained through the second terms in equations (2.5) and (2.7) that describe the information generation by infected individuals. Awareness is refreshed when infected individuals realize their condition (e.g. following a positive test result) changing their awareness level from their current value back to zero. This effect distinguishes between diseases with unmistakable symptoms and cases where the infection is contagious but asymptomatic, or where the infection might be mistaken for another condition. The parameter *ω* then varies not only from illness to illness, but it also depends on the mechanisms to identify the disease quickly, such as fast testing and contact-tracing programmes.

Finally, the *σI*_*Y*,*j*_ terms in equations (2.5)–(2.10) correspond to the recovery of infected individuals. The remaining terms correspond to the previously described information dynamics (see equations (2.1) and (2.2)), giving the changes in awareness level due to information exchange and fading. Summing over the information subscripts leads to

2.11 $$\frac{dS}{dt}=-\frac{\tilde{\beta }SI}{N},\;\frac{dI}{dt}=\frac{\tilde{\beta }SI}{N}-\sigma I\;\mathrm{and}\;\frac{dR}{dt}=\sigma I,$$

where

2.12 $$\tilde{\beta }(\rho ,\{S_{j},I_{i}\})=\beta \sum_{i,j=0}^{\infty }(1-\rho^{i})(1-\rho^{j})\frac{I_{i}}{I}\frac{S_{j}}{S},$$

yields an effective transmission rate due to protective measures taken by informed individuals. Here, *I*_*i*_ = *I*_*T*,*i*_ + *I*_*D*,*i*_ and *S*_*j*_ = *S*_*T*,*j*_ + *S*_*D*,*j*_ as before, and $I=\sum_{i}I_{i}$ and $S=\sum_{i}S_{i}$. This is similar to the SIR model, with *β* varying as the epidemic progresses. In fact, our model reduces to the SIR model if no awareness is present in the population and awareness generation is turned off, i.e. the population is distributed within subpopulations *X*_*Y*,∞_, and *ω* = 0, respectively. In the SIR model, the epidemic threshold is at *R*_0_ = *β*/*σ* = 1 [41]. In our model, the effective reproduction number has a similar form, $R_{e}=\tilde{\beta }/\sigma$. If we start with a fully uninformed and susceptible population, in which case awareness arises only through the process of information generation, then ${\tilde{\beta }}_{t=0}=\beta$, and the epidemic threshold is again at *R*_*e*_ = *β*/*σ*. Only if a certain level of awareness were already present in the population at the beginning of the epidemic, would the threshold be reduced to $R_{e}=\tilde{\beta }/\sigma$, where ${\tilde{\beta }}_{t=0}<\beta$.

Notice that no transmission would occur if the population was completely aware, i.e. with the best quality of information. This situation does not arise, however, because susceptibles can at best obtain an index 1 status if they are informed by infected individuals with first-hand information, *I*_0_. Additionally, a highly aware distrusting individual (resulting from either the distrusting individual becoming infected or predominant high-quality information in the population inhibiting the access to information of lower quality) may take a long time to obtain low-quality information if *α*_*D*_ is small and *λ* is large.

There is now mutual feedback between information and disease dynamics, where information is produced by newly infected individuals, increasing the level of awareness in the trusting population and eliciting higher levels of protection through a reduction in contacts and adherence to other non-pharmaceutical interventions, hindering the spread of the disease. With sufficiently fast spread of information in the trusting population, leading to a quicker response to an increase in the number of infections, a fine balance can be achieved. The presence of distrusting individuals in the population reduces the effectiveness of the protection generated by the trusting population, threatening this delicate equilibrium.

In the real world, the density of distrusting individuals could be estimated by polling the population about their willingness to follow guidelines provided by health agencies and the government, their trust in science and their goodwill to engage on self-initiated interventions to reduce transmission. Research developed during the current COVID-19 pandemic can be used to estimate this parameter. For example, Ipsos [42] estimates that 10% of the UK population believes that wearing face-covering in public is not important. Similarly, Wissenschaft im Dialog [43] suggests that 7–10% of the German population distrusts science and research. We assume that the composition of trusting and distrusting populations remains constant during the course of the epidemic. This assumption is supported by recent research that shows only limited changes to compliance with government rules and vaccine acceptance over time [44–46].

In the next section, we investigate the effects of varying the speed of information spread parameters, *α*_*T*_ and *α*_*D*_, the awareness-mediated protection parameter, *ρ*, and the density of distrusting individuals in the population, *d*, on the course and outcome of an epidemic.

## 3. Results

During an epidemic, the extent to which people comply with restrictions, such as mask-wearing, self-isolation and social distancing, and the effectiveness of such protective measures is pivotal to control the spread of the disease [47]. In our model, the parameters that capture these quantities are the awareness-mediated protection parameter *ρ*, and the density of distrusting individuals in the population, *d*.

### 3.1. The effects of varying *ρ* and *d*

Consider an initially unaware population composed of distrusting and trusting individuals with density *d* and 1 − *d*, respectively, where a small number of infected individuals are introduced in the population. Initially, there is no awareness in the population, and consequently no protection stemming from behavioural change. Hence, the disease spreads following a classic *SIR* model, where the threshold *R*_0_ = (*β*/*σ*) > 1 gives the condition for an initial increase in the number of cases.

As in the SIR model, if *R*_0_ < 1, the disease cannot spread in the population even in the absence of behavioural changes to control it. The awareness arising from the initial cases increases protection in the population and reduces the effective reproduction number *R*_*e*_ even further, accelerating the process of disease die out.

If *R*_0_ > 1, after an initial phase of unrestricted spread, the infected individuals will start to become aware of their condition, generating fresh information in the population. For a single infected individual, the time taken is of order *ω*^−1^. This awareness then spreads according to the information dynamics, eliciting protective measures among the informed individuals and causing a reduction in the effective reproduction number *R*_*e*_. If the response to new infections is sufficiently strong, a fine balance can be achieved, in which the increase in cases is counterbalanced by more cautious behaviour in the population. The presence of distrusting individuals, however, limits the effectiveness of the information-mediated protection in the population, as they will only comply with protective measures if directly affected by the disease.

**Figure 1.**
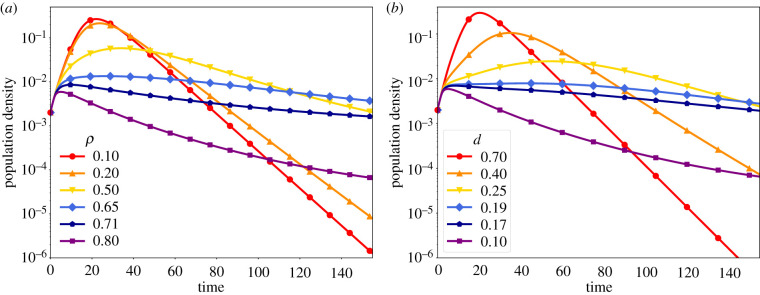
Stronger interventions (increasing *ρ*) have a similar effect on disease dynamics as a more trusting population (decreasing *d*). Curves show time series of the total number of infected *I*(*t*) for varying (*a*) *ρ* (fixed *d* = 0.1) and (*b*) *d* (fixed *ρ* = 0.8). In both cases *α*_*T*_ = *α*_*D*_ = 5, *β* = 0.667, *σ* = 0.133 (*R*_*e*_(0) = *R*_0_ = 5), *λ* = 0.2 and *ω* = 0.333.

In figure 1*a*, we show the trajectories of the total number of infected over time, *I*(*t*), for different *ρ* values, fixing the remaining parameters. Similarly, figure 1*b* shows *I*(*t*) for different distrusting densities *d*. We have chosen *R*_0_ > 1 so that we see non-trivial infection dynamics. In both cases, we observe an initial increase in the number of infected individuals that is common to all curves. This corresponds to the unaware phase with *R*_0_ > 1. After this initial phase of unrestricted contagion, there is a change in the curves’ behaviour as the parameters vary. For small *ρ* and large *d* values, the elicited protection is not able to suppress the outbreak after the initial contagion phase, in which case we observe an effect similar to a reduction in *β* for the SIR model, where the peak of infection occurs later, and the epidemic duration extends. By contrast, for large *ρ* values and small densities *d*, the evoked protective measures taken by the informed individuals are sufficient to bring and keep *R*_*e*_ below 1. The epidemic outbreak is then suppressed, and we observe a peak of infection happening earlier in the epidemic, also reducing its duration.

For intermediate values, we observe the delicate balance between new infections, generating awareness, and the reduction in transmissibility by the informed individuals. This leads to a plateau in the number of infected individuals in the population and to really long epidemics. A similar effect has been observed by Weitz *et al*. [11] in the context of an SEIR model without awareness spread, but including awareness-driven behavioural changes. There are critical values *ρ*_*c*_ and *d*_*c*_ for which we observe a phase transition in the behaviour of the epidemic. For *ρ* < *ρ*_*c*_ (or *d* > *d*_*c*_), the epidemic spread is only mitigated, while for *ρ* > *ρ*_*c*_ (*d* < *d*_*c*_) the outbreak is suppressed by the behavioural changes in the population. The value of *ρ*_*c*_ depends on the density of distrusting individuals in the population, and similarly *d*_*c*_ depends on the information-mediated protection levels. We investigate this dependence, *ρ*_*c*_ = *ρ*_*c*_(*d*), further in §3.2.

In the real world, resources such as hospital staff, ventilators, and ICU beds are limited. Thus, knowing how many people will be affected at the same time and require medical assistance is essential to manage epidemics. Not only that, being aware of the factors that influence the size of the peak of infection is extremely valuable to aid policymaking. To explore further the effects of *ρ* and *d* on the peak of infection, we solved equations (2.3)–(2.10) numerically for different values of *α* whilst again varying *ρ* and *d*.

**Figure 2.**
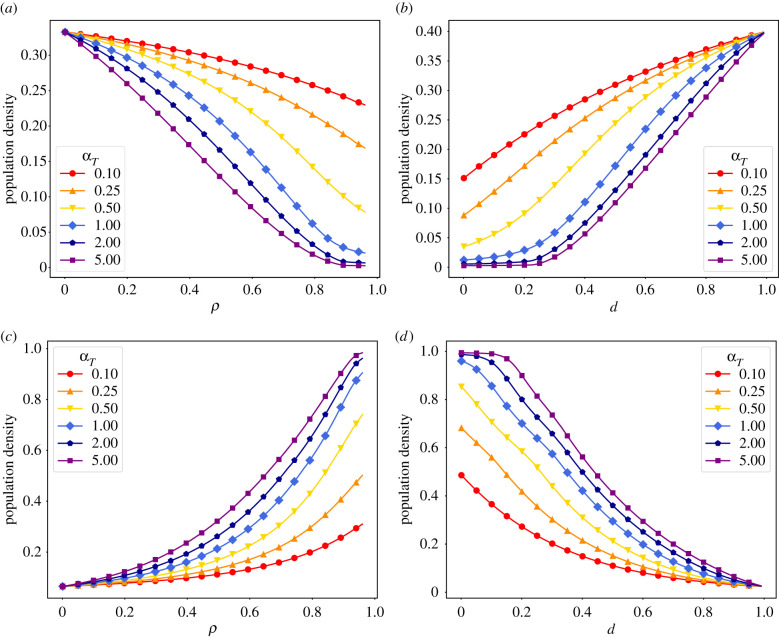
Faster information spread (increasing *α*) heightens model sensitivity to the strength of interventions (*ρ*) and composition of the population (*d*). Curves show effects of varying *ρ* and *d* on the peak infection size and the final susceptible population. (*a*) Total infections peak for varying *ρ* and fixed *d* = 0.3. (*b*) Total infections peak for fixed *ρ* = 0.8 and varying *d*. (*c*) Final susceptible population for varying *ρ* and fixed *d* = 0.3. (*d*) Final susceptible population for fixed *ρ* = 0.8 and varying *d*. Remaining parameters: *α*_*D*_ = 1, *λ* = 0.2, *ω* = 0.333, *β* = 0.667 and *σ* = 0.133.

Figure 2 shows the size of the peak of infection and the final susceptible population as a function of the parameters *ρ* and *d* for different *α*_*T*_ values. A common effect of behavioural feedback in epidemics is the reduction in the number of infected and increase in the final susceptible population [7,11,12]. We obtain a similar result in figure 2*a*,*c*. Increasing the information-mediated protection in the population, *ρ*, monotonically decreases the size of the peak of infection and hampers the spread of the disease, resulting in larger susceptible populations at the end of the epidemic.

By contrast, in figure 2*b*,*d* we observe that larger densities of distrusting individuals monotonically increase the size of the peak of infection and lead to more overall infections, resulting in smaller susceptible populations at the end of the epidemic. This stems from the reluctance that this portion of the population has to abide by protective measures.

**Figure 3.**
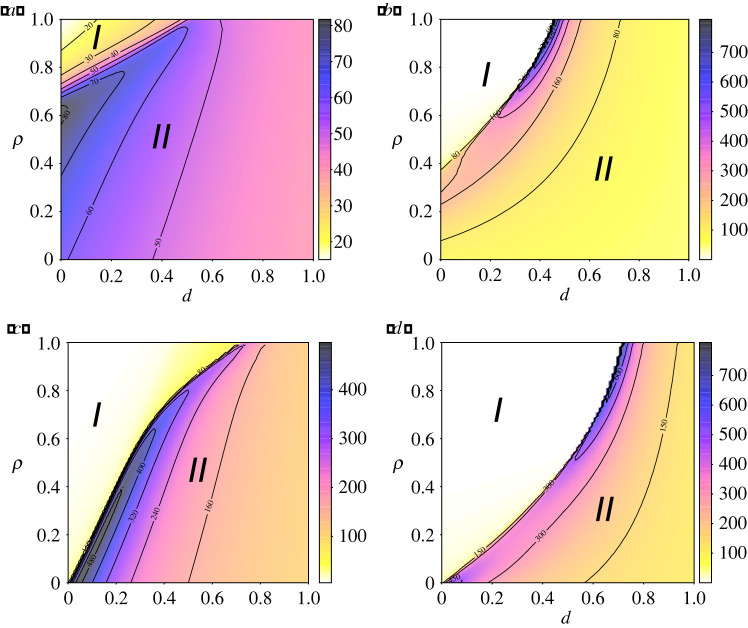
Timing of the infectious peak (colours) reveals the boundary between epidemic suppression and large outbreaks. First row: *R*_0_ = 3.5 (*β* = 0.467), (*a*) *α*_*T*_ = 0.5 and (*b*) *α*_*T*_ = 500. Second row: *R*_0_ = 2 (*β* = 0.267), (*c*) *α*_*T*_ = 0.5 and (*d*) *α*_*T*_ = 500. The remaining parameters are: *α*_*D*_ = 1, *λ* = 0.2, *ω* = 0.333 and *σ* = 0.133.

Other important disease outbreak metrics are the expected duration of an epidemic and the time when the peak of infection occurs. Figure 1 already gave us an idea of how these quantities vary with *ρ* and *d*. To get a better picture of their effect, we solved the system of equations numerically again and measured the time until the peak of infection for different combinations of *ρ* and *d*, and different *α*_*T*_ values. The results can be seen in figure 3. The initial condition consisted of an almost entirely susceptible, unaware population with initial infected population density *I*_*T*,∞_/*N* = *I*_*D*,∞_/*N* = 5 × 10^−6^.

The first thing to notice from figure 3 is the existence of the two regions mentioned earlier and the phase-transition, given by a curve *ρ*_*c*_(*d*). The first region (*I*), where *ρ* > *ρ*_*c*_(*d*), is the suppression regime, characterized by very low peaks of infection and times until the peak occurs that decrease as *ρ* increases. The second (*II*), where *ρ* < *ρ*_*c*_(*d*), is the mitigation regime, characterized by larger peaks of infection. In the mitigation regime, although the infection peak decreases as *ρ* increases, the times until the peak of infection increase, in contrast to the suppression regime. At the boundary, we observe a phase-transition, where the response to new infections is just strong enough to contain the increase in cases. At this boundary, we observe almost constant infection levels and, consequently, extended epidemics. The transition between the regions becomes more abrupt for larger *α*_*T*_ values, i.e. when awareness transmission is much faster in the trusting population.

In addition to the two regions, we note the existence of a critical value *d* = *d*_*M*_ above which suppression of the disease is not possible. This significant result highlights the need for effective measures to combat the spread of misinformation and the importance of maintaining trust in order to contain and suppress the disease. Furthermore, increasing the speed of information spread in the trusting population reduces *ρ*_*c*_(*d*), increasing the area of parameter space for which suppression is achievable, in contrast with the increase of *α*_*D*_, which increases *ρ*_*c*_(*d*), reducing the suppression region of the parameter space.

In essence, we observe a second threshold in the model. The first one corresponds to the outbreak threshold, where the disease can initially spread in the unaware population, given by *R*_0_ > 1. The second corresponds to the suppression threshold, where the protection elicited in the trusting population by the initial infection can reduce *R*_*e*_ to less than 1, hampering the spread of the disease. A second threshold due to preventive behaviour has been seen in the context of an SIS-like epidemiological model with awareness feedback on regular random networks and absence of fading mechanism [48], leading to a slow die-out of the epidemic and to a final population with a significant number of aware individuals. It has also been observed in [14,15] in the case of an SIRS model with two states of aware and unaware individuals. In [14,15], the authors observe that awareness changes the invasion conditions between a disease-free and endemic equilibrium, and can make it impossible for a disease to establish itself in the population. In both cases, the endemic equilibriums are characteristic of the chosen model, and the threshold can only be observed if awareness does not deteriorate through transmission or fading.

Naturally, the second threshold depends on the characteristics of the information spread dynamics, *α*_*T*_ and *α*_*D*_, the link between the effectiveness of behavioural change *ρ*, and the population composition, *d*. This threshold can be reflected in the effectiveness of protection elicited by behaviour change *ρ*_*c*_ given the population composition (density of distrusting individuals, *d*), and a fixed speed of information spread *α*_*T*_ and *α*_*D*_. Smaller *ρ*_*c*_ values mean that less protective measures are needed to reach the suppression threshold.

In the next section, we derive a theoretical expression for the value of *ρ*_*c*_ as function of *d* in the limit of fast information spread, i.e. large *α*_*T*_ and *α*_*D*_. In this case, we observe a separation of timescales between disease and information spread, allowing an analytical treatment of the simplified model.

### 3.2. Estimates of the critical *ρ* and density values under fast spread of information

Although it is difficult to express the suppression threshold analytically in most cases, as it depends on the distribution of awareness in the population and its complicated dynamics, we can make use of extra assumptions, such as fast information spread and large population size *N*, to simplify the model. The procedure is similar to obtaining the basic reproduction number in most epidemiological models [49].

Consider an initially unaware population of size *N* with a small density of infected individuals. Additionally, consider the limit in which the spread of information occurs almost instantaneously, i.e. *α*_*T*_ → ∞ and *α*_*D*_ → ∞. This would be the case, for instance, where new cases are informed globally through the internet or mass media as soon as they are discovered.

In this limit, there is a separation of timescales between the information and disease spread dynamics. A brief moment after the beginning of the epidemic, the first infected individual refreshes their information, moving to index 0. Immediately, all remaining trusting individuals in the higher index levels move to index 1, while all distrusting individuals remain unaware. Note that once infected distrusting individuals refresh information, they immediately become unaware again. We can then assume that the distrusting population occupies only the *S*_*D*,∞_, *I*_*D*,∞_ and *R*_*D*,∞_ subpopulations. In this scenario, the model simplifies to

3.1 $$\frac{dS_{T,1}}{dt}=-\frac{S_{T,1}}{N}(1-\rho )[(1-\rho )I_{T,1}+I_{D,\infty }]\beta ,$$

3.2 $$\frac{dS_{D,\infty }}{dt}=-\frac{S_{D,\infty }}{N}[(1-\rho )I_{T,1}+I_{D,\infty }]\beta ,$$

3.3 $$\frac{dI_{T,0}}{dt}=-\sigma I_{T,0}+\omega I_{T,1}-\lambda I_{T,0},$$

3.4 $$\begin{array}{rl} \frac{dI_{T,1}}{dt} & =-\sigma I_{T,1}-\omega I_{T,1}+\lambda I_{T,0}+\frac{S_{T,1}}{N}(1-\rho )[(1-\rho )I_{T,1}\\ & \;+I_{D,\infty }]\beta \end{array}$$

3.5 $$\mathrm{and}\;\frac{dI_{D,\infty }}{dt}=-\sigma I_{D,\infty }+\frac{S_{D,\infty }}{N}[(1-\rho )I_{T,1}+I_{D,\infty }]\beta ,$$

where we have omitted the equations for the removed populations. We drop the indices *T* and *D* for convenience as there is no shared information index between the two groups. As observed in the result section, we assume that the level of infected individuals is approximately constant (metastable) at the phase transition

3.6 $$\frac{dI}{dt}=\frac{dI_{0}}{dt}+\frac{dI_{1}}{dt}+\frac{dI_{\infty }}{dt}=0.$$

We also assume metastability of all three infected groups.

Setting d*I*_0_/d*t* = 0 gives *I*_0_ = (*ω*/(*σ* + *λ*))*I*_1_. Setting the right-hand side of equation (3.5) to zero, and assuming a large population such that, after the initial phase of unrestricted transmission, *S*_∞_/*N* ≈ *d*, where *d* is the initial density of distrusting individuals, and *S*_1_/*N* ≈ 1 − *d*, results in *I*_∞_ = [*d*(1 − *ρ*)/(*σ* − *βd*)]*βI*_1_. Now, from equation (3.6),

$$\begin{array}{rl} 0 & =-\frac{\sigma }{\beta }I_{1}\left(\frac{\omega }{\sigma +\lambda }+1+\frac{\beta d(1-\rho )}{\sigma -\beta d}\right)+(1-d){\left(1-\rho \right)}^{2}\left[1+\frac{\beta d}{\sigma -\beta d}\right]I_{1}\\ & \;+I_{1}d(1-\rho )\left[1+\frac{\beta d}{\sigma -\beta d}\right]. \end{array}$$

Denoting *x* = (1 − *ρ*) and *y* = *β*/(*σ* − *βd*), the previous equation becomes

3.7 $$\begin{array}{cc} & x^{2}(1-d)(1+dy)+x\;d\left(1+dy-\frac{y}{R_{0}}\right)-\frac{1}{R_{0}}\left(1+\frac{\omega }{\sigma +\lambda }\right)=0,\\ \mathrm{and}\;\mathrm{therefore}\;\;\; & x^{\pm }(d)=\frac{-d(1+dy-(y/R_{0}))\pm \sqrt{d^{2}{\left(1+dy-(y/R_{0})\right)}^{2}+(4/R_{0})(1+(\omega /\left(\sigma +\lambda \right)))(1-d)(1+dy)}}{2(1-d)(1+dy)}. \end{array}$$

We then obtain the critical protection level *ρ*_*c*_ as a function of the distrusting population density *d*, *ρ*_*c*_(*d*) = 1 − *x*(*d*). For *ρ* < *ρ*_*c*_(*d*) we observe mitigation of the disease spread, whilst for *ρ* > *ρ*_*c*_(*d*) the disease is suppressed.

From the expression above, we note that there is a critical distrusting density *d*_*M*_ = *σ*/*β* = 1/*R*_0_ above which suppression of the disease is not feasible irrespective of the level of information-mediated protection, *ρ*. Solutions for *d* > *d*_*M*_ yield negative *I*_∞_ population sizes and are, therefore, not physical. Similarly, we are interested in solutions for which 0 < *ρ* < 1, or equivalently, 0 < *x* < 1. Notice that, for *d* < *d*_*M*_, the *x*^+^ solution is always greater than zero, giving *ρ*_*c*_ < 1. Hence, there always exists a region for *d* < *d*_*M*_ in which suppression of the disease is achievable for sufficiently large *ρ*. Notice that, if *R*_0_ < 1, then *d*_*M*_ > 1. Hence, suppression is always achieved in this case. This is consistent with the threshold for an initial outbreak *R*_0_ > 1.

In particular, for *d* = 0,

3.8 $$\rho_{c}(0)=1-\sqrt{\frac{\sigma }{\beta }\left(1+\frac{\omega }{\sigma +\lambda }\right)},$$

gives us conditions where *ρ*_*c*_(0) > 0 and we can see a phase transition from mitigation to suppression for increasing *ρ* in the absence of distrusting individuals. If *ρ*_*c*_(0) < 0, then suppression is achieved for all *ρ* values, and we do not observe the transition as *ρ* increases. From equation (3.7), and more easily from equation (3.8), we can see that increasing *ω* while keeping the remaining parameters constant reduces *ρ*_*c*_(*d*), since more information is being produced and transmitted, which increases awareness in the population. By contrast, increasing *λ* increases *ρ*_*c*_(*d*) as the information is forgotten faster. Similarly, increasing *σ* while keeping *R*_0_ constant increases *ρ*_*c*_(*d*), as the probability that individuals recover before realizing they were infected increases.

**Figure 4.**
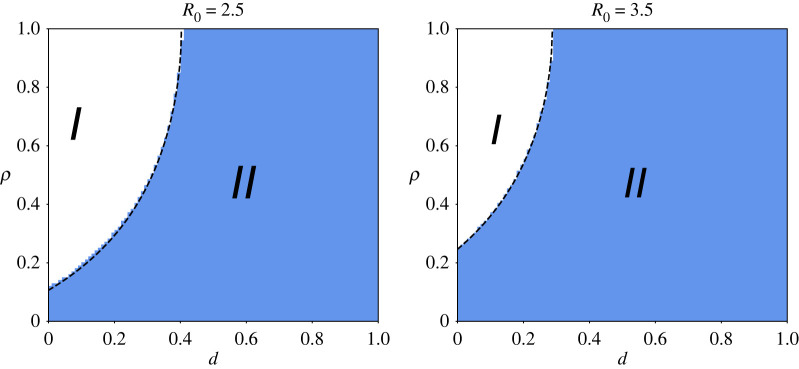
The agreement between the theoretical prediction (dashed lines) of the observed phase transition and the suppression/mitigation regions obtained through the numerical solutions of equations (2.3)–(2.10) for *α*_*T*_ = *α*_*D*_ = 10^6^, *λ* = 0.2, *ω* = 0.33, *σ* = 0.133, and (*a*) *β* = 0.333 (*R*_0_ = 2.5) and (*b*) *β* = 0.467 (*R*_0_ = 3.5). The white area (*I*) corresponds to the region where disease spread is suppressed. The area in blue (*II*) corresponds to the region where disease spread is only mitigated by behavioural feedback.

Figure 4 shows the regions of *d* − *ρ* parameter space corresponding to suppression (*I*) and mitigation (*II*), obtained through the numerical solution of the complete model (with large *α* and *I*_*T*,∞_/*N* = *I*_*D*,∞_/*N* = 5 × 10^−6^), and the theoretically predicted boundary (dashed black line), equation (3.7), for two values of *β* (and corresponding two values of *R*_0_). Good agreement was also observed for different combinations of *β* and *σ* (not shown), although discrepancies arise as the rate of information spread is reduced. Nonetheless, equation (3.7) shows the existence of regions of suppression and mitigation and a critical density of distrusting individuals, above which suppression is not achievable.

## 4. Discussion

Mathematical models are pivotal to policymaking and managing epidemics, giving insight into important aspects of the dynamics of a disease that can facilitate its control and predict possible outcomes. In the past, epidemiological models failed to account for the important aspect of behavioural responses to diseases in human populations. More recently, prominent approaches focusing on the spread of awareness and its interplay with disease dynamics have been developed [5].

In this article, we developed a model that merged epidemiological dynamics with information spread and behavioural change interactions of both trusting and distrusting individuals in a population. This model is an extension of previous work in this area which considered only one class of individuals [12]. Using our model, we were able to highlight the mechanisms by which the burden of epidemics can be lightened.

Our results show that the surge in awareness, arising from fast spread of information in the trusting population, can reduce the peak of infection and lead to a larger population that remains uninfected by the end of the outbreak. A similar effect is observed by increasing the effectiveness of protective measures taken by well-informed individuals. We also observed a phase transition between mitigation and suppression regimes. In the mitigation regime, the behavioural reaction and reduction in transmissibility arising in the population is not sufficient to reduce the effective reproduction number below one. Hence, the observed effect in the infection curve is a lower and delayed peak. However, if the response to new cases is sufficiently strong, a fine balance between new infections and a reduction in transmissibility can be achieved. In this case, we observe an almost constant level of infection over time, leading to extended epidemics. We then observe a phase transition, in which further increasing the effectiveness of the measures to control the spread of the disease leads to a regime of suppression. By contrast, larger densities of distrusting individuals in the population limit the effectiveness of the reduced transmissibility in the trusting population, resulting in the existence of a critical density *d*_*M*_ from which the suppression regime is not achievable.

Close to the phase-boundary there is a non-trivial benefit to be gained. Even the slightest changes in the density of distrusting population or degree of adherence to NPIs can be the difference between suppressing an outbreak or merely mitigating against it. This highlights the importance of efficient information dissemination in the trusting population, the adoption of effective non-pharmaceutical interventions, and the urgent need to better inform the public and increase compliance with precautionary measures as a means to reduce disease spread and lighten the disease-associated healthcare burden. The WHO has published reports on how to manage current and future infodemics [50,51]. Among the recommended measures to tackle these problems, they suggest improvements in education (especially science education, numeracy and critical thinking), and the identification of the groups most vulnerable to misinformation for which action should be prioritized. In [23], it is suggested that governments provide clear information via official channels to reduce public anxiety and increase trust, as well as taking action against the spread of false information. It also suggests that social media platforms should apply more stringent censorship of pandemic-related misinformation.

Although a complete and simple analytical characterization of the suppression threshold is difficult in general, as it depends on the complex dynamics of the system, we were able to write an expression for *ρ*_*c*_(*d*) in the limit of fast information transmission and assuming a large population size. The expression agreed with the numerically observed boundary in the corresponding parameter regime, and allowed us to obtain the maximum proportion of distrusting individuals for which suppression of the outbreak is achievable. A possibly fruitful direction for further research to obtain approximations for realistic information transmission parameter values, but that still exhibit slow and fast dynamics, is the analysis of the stochastic version of the system using the framework developed in [52] that allows for dimension reduction in stochastic dynamical systems with a separation of timescales. The method is exact in the limit of well-separated fast-and-slow dynamics and small noise, and found to be a reasonable approximation over a sensible parameter range.

In our model, the density of distrusting individuals, *d*, has been assumed to be constant throughout the outbreak, while personal attitudes such as handwashing, the use of face-coverings and social distancing, may change according to the state of the pandemic, reflecting changes in awareness and risk perception of the population as the number of new cases increases or decreases. Studies developed during the COVID-19 pandemic show the low extent to which compliance with government rules and vaccine acceptance changed over time [44–46] supporting our assumption. Relaxing this assumption to include possible opinion shifts is a noteworthy extension to our model.

Moreover, the kind of misinformation that may push people towards the distrusting group has its own dynamics [53] and is a fascinating research topic by itself [54,55]. Despite its importance, it is still a developing area of research with many prospective directions for future work. Shifts in opinions may come about as a result of information transmission interactions between individuals in the population, or as the average of the opinions of the nodes’ neighbours in a network. More realistic models may include mechanisms such as homophily [56] and opinion amplification [7].

Another limitation of the model developed here is the underlying assumption of a well-mixed population, for which the explicit spatial structure of the system is disregarded. Previous work on the effects of spatial structure in the spread of infectious diseases has shown its importance in the invasion threshold for epidemics [57], and the development of control policies [58–61]. The analysis of epidemiological models with awareness feedback on networks with homogeneous populations has been an active area of research [9,12,62–67]. However, these models disregard the heterogeneous behaviour presented here. Thus, the inclusion of spatial dynamics in our model is another prospective direction for further work.

Hitherto, we have considered only the adoption of non-pharmaceutical interventions, which do not change the disease state of individuals. Yet, it has been shown that vaccination intention has been highly correlated with trust in the government and exposure to misinformation [37–39]. As another prospective direction for future research, we aim to extend our modelling framework to include vaccination hesitance. In [68], the authors develop a model to explore the intricate dynamics of three contagion processes: the transmission of disease, fear of contagion and fear of vaccination. Their work reveals mechanisms for the emergence of multiple waves; their size, shape and form. Moreover, in [69], the authors model anti-vaccine sentiment as a cultural pathogen. They find that sentiment dynamics may lead to a sudden return of the disease after a long period of dormancy and to endemicity when it would have disappeared otherwise. These studies draw attention to the importance of research on the topic and the development of increasingly realistic behavioural models to give better insight on the management of epidemics.

## Data Availability

Code is publicly available at https://github.com/amsontag/mcpse.

## References

[1] Hays JN. 2006 Epidemics and pandemics: their impacts on human history. Santa Barbara, CA: ABC-CLIO.

[2] Howard J et al. 2021 An evidence review of face masks against COVID-19. Proc. Natl Acad. Sci. USA **118**, e2014564118. (10.1073/pnas.2014564118) 33431650 PMC7848583

[3] Derek K et al. 2020 Physical distancing, face masks, and eye protection to prevent person-to-person transmission of SARS-CoV-2 and COVID-19: a systematic review and meta-analysis. Lancet **395**, 1973-1987. (10.1016/S0140-6736(20)31142-9) 32497510 PMC7263814

[4] Mahase E. 2020 Covid-19 was the decision to delay the UK’s lockdown over fears of ‘behavioural fatigue’ based on evidence? Br. Med. J. **370**, m3166. (10.1136/bmj.m3166) 32769080

[5] Funk S, Salathé M, Jansen VAA. 2010 Modelling the influence of human behaviour on the spread of infectious diseases: a review. J. R. Soc. Interface **7**, 1247-1256. (10.1098/rsif.2010.0142) 20504800 PMC2894894

[6] Reluga TC. 2010 Game theory of social distancing in response to an epidemic. PLoS Comput. Biol. **6**, e1000793. (ed. CT Bergstrom). (10.1371/journal.pcbi.1000793) 20523740 PMC2877723

[7] Tyson RC, Hamilton SD, Lo AS, Baumgaertner BO, Krone SM. 2020 The timing and nature of behavioural responses affect the course of an epidemic. Bull. Math. Biol. **82**, 14. (10.1007/s11538-019-00684-z) 31932981 PMC7223272

[8] Epstein JM, Parker J, Cummings D, Hammond RA. 2008 Coupled contagion dynamics of fear and disease: mathematical and computational explorations. PLoS ONE **3**, e3955. (10.1371/journal.pone.0003955) 19079607 PMC2596968

[9] Rizzo A, Frasca M, Porfiri M. 2014 Effect of individual behavior on epidemic spreading in activity-driven networks. Phys. Rev. E **90**, 042801. (10.1103/PhysRevE.90.042801) 25375543

[10] Kabir KMA, Kuga K, Tanimoto J. 2019 Analysis of SIR epidemic model with information spreading of awareness. Chaos Solitons Fractals **119**, 118-125. (10.1016/j.chaos.2018.12.017)

[11] Weitz JS, Park SW, Eksin C, Dushoff J. 2020 Awareness-driven behavior changes can shift the shape of epidemics away from peaks and toward plateaus, shoulders, and oscillations. Proc. Natl Acad. Sci. USA **117**, 32 764-32 771. (10.1073/pnas.2009911117) PMC7768772 33262277

[12] Funk S, Gilad E, Watkins C, Jansen VAA. 2009 The spread of awareness and its impact on epidemic outbreaks. Proc. Natl Acad. Sci. USA **106**, 6872-6877. (10.1073/pnas.0810762106) 19332788 PMC2672559

[13] Allen LJS. 2017 A primer on stochastic epidemic models: formulation, numerical simulation, and analysis. Infect. Dis. Model. **2**, 128-142. (10.1016/j.idm.2017.03.001) 29928733 PMC6002090

[14] Funk S, Salathé M, Jansen VAA. 2010 Modelling the influence of human behaviour on the spread of infectious diseases: a review. J. R. Soc. Interface **7**, 1247-1256. (10.1098/rsif.2010.0142) 20504800 PMC2894894

[15] Kiss IZ, Cassell J, Recker M, Simon PL. 2010 The impact of information transmission on epidemic outbreaks. Math. Biosci. **225**, 1-10. (10.1016/j.mbs.2009.11.009) 19948177

[16] Hornsey MJ, Harris EA, Fielding KS. 2018 The psychological roots of anti-vaccination attitudes: a 24-nation investigation. Health Psychol. **37**, 307-315. (10.1037/hea0000586) 29389158

[17] King's College London. 2020 The three groups reacting to life under lockdown. See https://www.kcl.ac.uk/news/the-three-groups-reacting-to-life-under-lockdown.

[18] King’s College London. 2021 Trust in lockdown sceptics linked to arguments about COVID vaccine See https://www.kcl.ac.uk/news/trust-in-lockdown-sceptics-linked-to-arguments-about-covid-vaccine.

[19] Murphy J. 2021 Psychological characteristics associated with COVID-19 vaccine hesitancy and resistance in Ireland and the United Kingdom. Nat. Commun. **12**, 29. (10.1038/s41467-020-20226-9) 33397962 PMC7782692

[20] Dordević JM, Mari S, Vdovic M, Milošević A. 2021 Links between conspiracy beliefs, vaccine knowledge, and trust: anti-vaccine behavior of Serbian adults. Soc. Sci. Med. **277**, 113930. (10.1016/j.socscimed.2021.113930) 33873008 PMC8634900

[21] Farooq F, Rathore FA. 2021 COVID-19 vaccination and the challenge of infodemic and disinformation. J. Korean Med. Sci. **36**, e78. (10.3346/jkms.2021.36.e78) 33724740 PMC7961870

[22] Naeem SB, Boulos MNK. 2021 COVID-19 misinformation online and health literacy: a brief overview. Int. J. Environ. Res. Public Health **18**, 8091. (10.3390/ijerph18158091) 34360384 PMC8345771

[23] Pian W, Chi J, Ma F. 2021 The causes, impacts and countermeasures of COVID-19 ‘Infodemic’: a systematic review using narrative synthesis. Inf. Process. Manage. **58**, 102713. (10.1016/j.ipm.2021.102713) PMC8545871 34720340

[24] Pool J, Fatehi F, Akhlaghpour S. 2021 Infodemic, misinformation and disinformation in pandemics: scientific landscape and the road ahead for public health informatics research, vol. 281, pp. 764-768. Studies in Health Technology and Informatics. Amsterdam, Netherlands: IOS Press. 10.3233/SHTI210278 34042681

[25] W.H. Organization, Infodemic. See https://www.who.int/health-topics/infodemic#tab=tab_1 (accessed 1 March 2022).

[26] Islam MS et al. 2020 COVID-19–related infodemic and its impact on public health: a global social media analysis. Am. J. Trop. Med. Hyg. **103**, 1621-1629. (10.4269/ajtmh.20-0812) 32783794 PMC7543839

[27] Zarocostas J. 2020 How to fight an infodemic. Lancet **395**, 676. (10.1016/S0140-6736(20)30461-X) 32113495 PMC7133615

[28] Vosoughi S, Roy D, Aral S. 2018 The spread of true and false news online. Science **359**, 1146-1151. (10.1126/science.aap9559) 29590045

[29] Banerjee D, Meena KS. 2021 COVID-19 as an ‘infodemic’ in public health: critical role of the social media. Front. Public Health **9**, 231. (10.3389/fpubh.2021.610623) PMC8012664 33816415

[30] DeJong C, Wachter RM. 2020 The risks of prescribing hydroxychloroquine for treatment of COVID-19—first, do no harm. JAMA Intern. Med. **180**, 1118-1119. (10.1001/jamainternmed.2020.1853) 32347894

[31] Allington D, Duffy B, Wessely S, Dhavan N, Rubin J. 2020 Health-protective behaviour, social media usage and conspiracy belief during the COVID-19 public health emergency. Psychol. Med. **51**, 1763-1769. (10.1017/S003329172000224X) 32513320 PMC7298098

[32] Banai IP, Banai B, Mikloušić I. 2021 Beliefs in COVID-19 conspiracy theories, compliance with the preventive measures, and trust in government medical officials. Curr. Psychol. https://link.springer.com/article/10.1007/s12144-021-01898-y. 10.1007/s12144-021-01898-y PMC8153526 34075284

[33] Bernard R, Bowsher G, Sullivan R, Gibson-Fall F. 2021 Disinformation and epidemics: anticipating the next phase of biowarfare. Health Secur. **19**, 3-12. (10.1089/hs.2020.0038) 33090030 PMC9195489

[34] Hovland CI, Weiss W. 1951 The influence of source credibility on communication effectiveness. Public Opin. Q. **15**, 635-650. (10.1086/266350)

[35] Quinn SC, Parmer J, Freimuth VS, Hilyard KM, Musa D, Kim KH. 2013 Exploring communication, trust in government, and vaccination intention later in the 2009 H1N1 pandemic: results of a national survey. Biosecur. Bioterror. **11**, 96-106. (10.1089/bsp.2012.0048) 23617721 PMC3689165

[36] Dubé E, Laberge C, Guay M, Bramadat P, Roy R, Bettinger JA. 2013 Vaccine hesitancy. Hum. Vaccine Immunother. **9**, 1763-1773. (10.4161/hv.24657) PMC3906279 23584253

[37] Jolley D, Douglas KM. 2014 The effects of anti-vaccine conspiracy theories on vaccination intentions. PLoS ONE **9**, e89177. (10.1371/journal.pone.0089177) 24586574 PMC3930676

[38] Islam MS et al. 2021 COVID-19 vaccine rumors and conspiracy theories: the need for cognitive inoculation against misinformation to improve vaccine adherence. PLoS ONE **16**, e0251605. (10.1371/journal.pone.0251605) 33979412 PMC8115834

[39] Loomba S, de Figueiredo A, Piatek SJ, de Graaf K, Larson HJ. 2021 Measuring the impact of COVID-19 vaccine misinformation on vaccination intent in the UK and USA. Nat. Hum. Behav. **5**, 337-348. (10.1038/s41562-021-01056-1) 33547453

[40] Anderson RM, May RM. 1991 Infectious diseases of humans, dynamics and control. Oxford: Oxford University Press.

[41] Diekmann O, Heesterbeek JAP. 2000 Mathematical epidemiology of infectious diseases: model building, analysis and interpretation. New York, NY: John Wiley & Sons.

[42] Ipsos MORI. 2020 Nearly 9 in 10 britons say it is either essential or important to wear a facemask in public to stop the spread of coronavirus. See https://www.ipsos.com/ipsos-mori/en-uk/nearly-9-10-britons-say-it-either-essential-or-important-wear-facemask-public-stop-spread (accessed on 01/03/2022).

[43] Wissenschaft im Dialog. Science barometer 2020. See https://www.wissenschaft-im-dialog.de/en/our-projects/science-barometer/science-barometer-2020/.

[44] Ipsos MORI. 2021 Most Britons continue to say they are following coronavirus rules; almost half believe lockdown measures are not strict enough. See https://www.ipsos.com/ipsos-mori/en-uk/most-britons-continue-say-they-are-following-coronavirus-rules-almost-half-believe-lockdown (accessed on 03/01/2022).

[45] Ipsos MORI. Half say they would be very likely to follow instructions to self-isolate if pinged. See https://www.ipsos.com/ipsos-mori/en-uk/half-say-they-would-be-very-likely-follow-instructions-self-isolate-if-pinged (accessed on 03/01/2022).

[46] Hamel L, Lopes L, Sparks G, Kirzinger A, Kearney A, Stokes M, Brodie M. 2021 KFF COVID-19 vaccine monitor: October 2021. See https://www.kff.org/coronavirus-covid-19/poll-finding/kff-covid-19-vaccine-monitor-october-2021/ (accessed on 01/03/2022).

[47] Catching A, Capponi S, Yeh MT, Bianco S, Andino R. 2021 Examining the interplay between face mask usage, asymptomatic transmission, and social distancing on the spread of COVID-19. Sci. Rep. **11**, 15998. (10.1038/s41598-021-94960-5) 34362936 PMC8346500

[48] Juher D, Kiss IZ, Saldaña J. 2015 Analysis of an epidemic model with awareness decay on regular random networks. J. Theor. Biol. **365**, 457-468. (10.1016/j.jtbi.2014.10.013) 25452138

[49] van den Driessche P, Watmough J. 2002 Reproduction numbers and sub-threshold endemic equilibria for compartmental models of disease transmission. Math. Biosci. **180**, 29-48. (10.1016/S0025-5564(02)00108-6) 12387915

[50] World Health Organization. 2020 An ad hoc WHO technical consultation managing the COVID-19 infodemic: call for action, 7-8 April 2020, vol. **viii**. See https://apps.who.int/iris/handle/10665/334287.

[51] World Health Organization. 2021 WHO public health research agenda for managing infodemics. See https://www.who.int/publications/i/item/9789240019508

[52] Parsons TL, Rogers T. 2017 Dimension reduction for stochastic dynamical systems forced onto a manifold by large drift: a constructive approach with examples from theoretical biology. J. Phys. A: Math. Theor. **50**, 415601. (10.1088/1751-8121/aa86c7)

[53] Grimes DR. 2021 Medical disinformation and the unviable nature of COVID-19 conspiracy theories. PLoS ONE **16**, e0245900. (10.1371/journal.pone.0245900) 33711025 PMC7954317

[54] Tambuscio M, Ruffo G, Flammini A, Menczer F. 2015 Fact-checking effect on viral hoaxes. In *WWW '15 Companion: Proc. of the 24th Int. Conf. on World Wide Web. Florence, Italy, 18–22 May 2015*. New York, NY: Association for Computing Machinery. (10.1145/2740908.2742572)

[55] Tambuscio M, Oliveira DFM, Ciampaglia GL, Ruffo G. 2018 Network segregation in a model of misinformation and fact-checking. J. Comput. Soc. Sci. **1**, 261-275. (10.1007/s42001-018-0018-9)

[56] Aiello LM, Barrat A, Schifanella R, Cattuto C, Markines B, Menczer F. 2012 Friendship prediction and homophily in social media. ACM Trans. Web **6**, 1-33. (10.1145/2180861.2180866)

[57] Keeling MJ. 1999 The effects of local spatial structure on epidemiological invasions. Proc. R. Soc. Lond. B **266**, 859-867. (10.1098/rspb.1999.0716) PMC1689913 10343409

[58] Keeling MJ et al. 2001 Dynamics of the 2001 UK foot and mouth epidemic: stochastic dispersal in a heterogeneous landscape. Science **294**, 813-817. (10.1126/science.1065973) 11679661

[59] Ferguson NM, Donnelly CA, Anderson RM. 2001 Transmission intensity and impact of control policies on the foot and mouth epidemic in Great Britain. Nature **413**, 542-548. (10.1038/35097116) 11586365

[60] Ferguson NM, Cummings DAT, Cauchemez S, Fraser C, Riley S, Meeyai A, Iamsirithaworn S, Burke DS. 2005 Strategies for containing an emerging influenza pandemic in Southeast Asia. Nature **437**, 209-214. (10.1038/nature04017) 16079797

[61] Gudelj I, White KAJ. 2004 Spatial heterogeneity, social structure and disease dynamics of animal populations. Theor. Popul. Biol. **66**, 139-149. (10.1016/j.tpb.2004.04.003) 15302223

[62] Bansal S, Grenfell BT, Meyers LA. 2007 When individual behaviour matters: homogeneous and network models in epidemiology. J. R. Soc. Interface **4**, 879-891. (10.1098/rsif.2007.1100) 17640863 PMC2394553

[63] Sahneh FD, Scoglio CM. 2012 Optimal information dissemination in epidemic networks. In *2012 IEEE 51st IEEE Conf. on Decision and Control (CDC), Maui, HI, USA, 10--13 December 2012*. Piscataway, NJ: IEEE.

[64] Granell C, Gómez S, Arenas A. 2013 Dynamical interplay between awareness and epidemic spreading in multiplex networks. Phys. Rev. Lett. **111**, 128701. (10.1103/PhysRevLett.111.128701) 24093306

[65] Sahneh FD, Chowdhury FN, Brase G, Scoglio CM. 2014 Individual-based information dissemination in multilayer epidemic modeling. Math. Model. Nat. Phenomena **9**, 136-152. (10.1051/mmnp/20149209)

[66] Kan J-Q, Zhang H-F. 2017 Effects of awareness diffusion and self-initiated awareness behavior on epidemic spreading—an approach based on multiplex networks. Commun. Nonlinear Sci. Numer. Simul. **44**, 193-203. (10.1016/j.cnsns.2016.08.007) 32288421 PMC7128930

[67] Hu P, Ding L, An X. 2018 Epidemic spreading with awareness diffusion on activity-driven networks. Phys. Rev. E **98**, 062322. (10.1103/PhysRevE.98.062322)

[68] Epstein JM, Hatna E, Crodelle J. 2021 Triple contagion: a two-fears epidemic model. J. R. Soc. Interface **18**, 20210186. (10.1098/rsif.2021.0186) 34343457 PMC8331242

[69] Mehta RS, Rosenberg NA. 2020 Modelling anti-vaccine sentiment as a cultural pathogen. Evol. Hum. Sci. **2**, e21. (10.1017/ehs.2020.17) PMC10427458 37588376

